# Looking beyond electroconvulsive therapy: A case report

**DOI:** 10.1192/j.eurpsy.2021.2068

**Published:** 2021-08-13

**Authors:** D. Barbosa, A. Sousa, A.S. Machado, R. Moreira, M. Mota

**Affiliations:** Psychiatry, Sao Joao Hospital and University Centre, Porto, Portugal

**Keywords:** Electroconvulsive therapy, Depression, treatment response

## Abstract

**Introduction:**

Electroconvulsive therapy (ECT) is considered a gold-standart treatment of severe and treatment-resistant depression. Lack of response to ECT often causes distress in psychiatrists regarding the next therapeutic decisions.

**Objectives:**

To present a case report of a patient with psychotic depression with partial response to ECT.

**Methods:**

Clinical interviews and review of literature using the Pubmed platform.

**Results:**

The authors present a case of a 60 year-old woman admitted for severe depressive episode with psychotic symptoms. Due to lack of response to multiple antidepressive and antipsychotic treatments, 15 sessions of ECT were performed with improvement of behavioral and psychotic symptoms. However, endogenous depressive symptoms with functional impairment persisted. It was then initiated Bupropion 300mg/day resulting in vast improvements on drive, energy and activity levels with restored functionality. Previously to ECT, Bupropion was not considered a valid option due to the psychomotor restlessness that was present. This case exposes the limitations of ECT and the therapeutic conundrums that arise when there is partial response. The symptoms expressed in the patient after ECT course correlate with deficits in noradrenergic and dopaminergic pathways that are involved in endogenous depression. The use of Bupropion, with its effect on noradrenaline and dopamine receptors, may offer a therapeutic lifeline in these cases.

**Conclusions:**

ECT still stands as a gold-standart for severe depressive disorder, especially when several psychopharmacological therapies have failed. In cases of partial response to ECT, the neurobiological correlates of clinical presentation can guide the therapeutic management towards improved outcomes.

**Disclosure:**

No significant relationships.

